# New Parameters Extracted from Tilted Fiber Bragg Grating Spectra for the Determination of the Refractive Index and Cut-Off Wavelength

**DOI:** 10.3390/s19091964

**Published:** 2019-04-26

**Authors:** Sławomir Cięszczyk, Piotr Kisała, Janusz Mroczka

**Affiliations:** 1 Institute of Electronics and Information Technology, Lublin University of Technology, 20-618 Lublin, Poland; s.cieszczyk@pollub.pl; 2 Electronic and Photonic Metrology, Wroclaw University of Technology, 50-317 Wroclaw, Poland; janusz.mroczka@pwr.wroc.pl

**Keywords:** fiber-optic sensors, TFBG, demodulation method, refractive index

## Abstract

Tilted fiber Bragg grating (TFBG) is a very popular fiber optic element that is used as a sensor for various physical quantities. The calculation of the refractive index of a substance surrounding the TFBG is based on its spectrum demodulation, which consists of determining a certain parameter that is correlated with the sought quantity. The most commonly used parameter is the area created by the maxima and minima of the cladding mode resonances. In this article, we propose a new group of methods, which are based on calculating the parameters related to the spectrum differences between the local average values in the range of occurrence of the cladding modes. The basic parameter used in this group of methods is the mean absolute deviation from the local mean, which is characterized by the best linearity among the considered group of methods. The calculated parameters, in their cumulative form, can also be used to determine the cut-off wavelength, which can also indirectly indicate the refractive index value. The proposed approaches were compared, in terms of measurement resolution, to the most commonly used methods, such as the cladding modes’ envelope area and the spectral contour lengths.

## 1. Introduction

**Figure 1:**
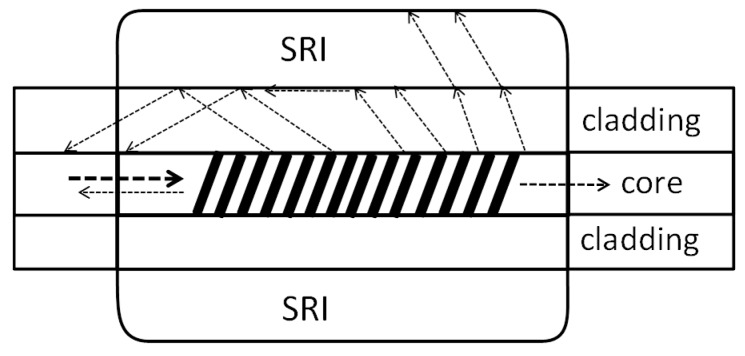
Diagram of a tilted fiber Bragg grating (TFBG)-based refractometer.

The refractive index is an important parameter, which is often subject to monitoring in many chemical and biological processes. Optical fiber sensors, including Bragg gratings, can be used to determine the refractive index value. The basic advantage of fiber optics is their small size and low invasiveness. Classic fiber Bragg gratings (FBGs) are not sensitive to the surrounding refractive index (SRI), because the light propagated in the core penetrates only a small depth of the cladding. SRI sensitivity can be obtained by removing the cladding from the fiber, among other methods. These sensors are characterized by high temperature cross-sensitivity [1]. An alternative method is the use of recently developed sensors based on tilted fiber Bragg gratings (TFBGs). In this type of structure, so-called cladding modes appear, which are associated with the discrete backward coupling of light from the core to the cladding (Figure 1). A large number of cladding modes result from the large size of the cladding. The cladding modes are strongly influenced by the surrounding medium. They propagate close to the border of the cladding; therefore, the SRI affects their effective refractive index. The cladding modes propagate in the opposite direction from the light propagation in the core. In the transmission spectrum, they are visible in the form of a fine comb. There are two other peaks in the transmission spectrum: the main core mode and the ghost mode. For SRI measurement, only cladding modes can be used. They are not visible in the reflection spectrum as a result of the high suppression of the modes in the fiber cladding. The individual cladding modes have a varied sensitivity to changes in external conditions. High-order cladding modes are more sensitive to changes in the SRI. The cladding modes exposed to increases in the SRI go into leaky modes, during which they do not reflect through the total internal reflection at the border of the fiber cladding and the surrounding refractive index. This means that their effective refractive index is larger than the surrounding refractive index.

TFBG sensitivity to a specific SRI range can be selected by the tilt angle of the grating planes. It is also possible to modify the sensitivity range by changing the diameter of the optical fiber. For example, when this diameter is reduced, sensitivity to the SRI increases [2]. There are also interesting modifications such as multi-angle TFBG, containing several gratings, one after the other, with varying tilt angles [3]. Additional TFBG-based sensor sensitivity can be increased by two main methods. In the first method, the fiber with the grating must be covered with special materials to achieve a plasmonic effect. The second method requires the use of spectral measurements with strictly controlled polarizations [4,5]. However, this requires additional measurement of the spectrum for several polarizations, which complicates the sensor setup. In most applications, it is better to use a simple experimental setup. Good measurement properties can be achieved by appropriately selecting the spectra processing, which is the aim of this study.

Changes in the TFBG spectrum caused by changes in the medium surrounding the grating are known and described in many publications. There are also known methods for determining parameters based on measured spectra, which are directly related to the SRI. We propose a new group of methods based on the parameters calculated as a deviation from the local mean of the spectrum and cumulative parameters for cut-off mode determination. Moreover, we compared our new proposed methods with the existing algorithms in the literature.

## 2. Methods for SRI Determination Based on TFBG Spectrum Analysis

SRI measurements using TFBG sensors rely generally on the extraction of specific parameters of the TFBG spectra that are correlated with the SRI. Then, using calibration measurements, the relationship between the extracted parameters and the SRI is determined. Based on the literature, the existing methods of spectrum demodulation can be divided as follows:

(a) Determination of the wavelength shifts of individual cladding modes [6];
(b) Determination of the cut-off length of the cladding modes [7,8,9,10];
(c) Determination of the parameters of transmission spectrum changes in the range of cladding modes [11,12,13].

The method for determining the wavelength shift of individual cladding modes can only be applied to a narrow SRI range. The processing characteristics between the SRI and the wavelength shift are strongly non-linear, and the sensitivity increases with the increasing SRI until the mode disappears. The temperature insensitivity of this method can be achieved by simultaneously monitoring the main shift of the Bragg resonance [6]. It can be assumed that the shifts of the cladding modes and the core mode have the same temperature values. This method is also used to determine the refractive index by estimating the wavelength shifts of the transmission dips of slanted multimode fiber Bragg grating [14].

Changes in the cladding mode wavelength position due to SRI changes for small SRIs are minor at 20 pm/u.r.i. and grow rapidly with the increasing SRI up to 100 times. This is because the mode, which is close to being cut off, propagates deeper into the surrounding medium. In order to obtain high measurement sensitivity with respect to the refractive index changes, it is necessary to choose modes close to the cut-off limit. However, due to the high non-linearity of the relationship between the wavelength position and the SRI, the determination of this dependence requires a large number of calibration points. The other two types of methods can be considered to be global methods, because they use the spectral range of all the cladding modes and are capable of determining the widest range of SRI for a given type of grating. Probably the most popular method, which allows the spectrum changes and SRI changes to be quantitatively correlated, is to monitor the areas limited by the cladding modes. In this method, the area formed by the lower and upper envelopes is monitored, and its value decreases as the SRI increases [13]:

(1) $$A_{E}=\frac{\int_{\lambda_{\min}}^{\lambda_{\max}}\left[T_{up}\left(\lambda \right)-T_{low}\left(\lambda \right)\right]d\lambda }{\int_{\lambda_{\min}}^{\lambda_{\max}}\left[T_{up}^{ref}\left(\lambda \right)-T_{low}^{ref}\left(\lambda \right)\right]d\lambda }$$

where *T_up_* and *T_low_* are the upper and lower cladding mode envelopes. The transmission values in the denominator with the refractive indexes are used for normalization and most frequently represent values for a clear water refractive index. An extension of this method is the use of skewness and kurtosis parameters calculated from the spectrum to increase the range of SRI determination below the SRI of pure water [11]. This method, calculating the area of the TFBG cladding modes’ envelopes, is also used for liquid level monitoring [15]. The SRI can also be indicated by the transmission spectrum area changes, defined as follows [16]:

(2) $$A_{TS}=\int_{\lambda_{\min}}^{\lambda_{\max}}T\left(\lambda \right)d\lambda$$

where *λ_min_* and *λ_max_* are the wavelengths of the beginning and end of the considered range of cladding modes’ transmission spectra T(λ).

Transmission changes in the cladding modes’ spectral range can also indicate the SRI value [2]. These changes may be measured directly by the power of the light propagating through the TFBG. In addition to changes in the transmission, the power of radiation sources can also be measured as the reference [17]. TFBG combined with an additional chirped grating covering the entire range of cladding modes can be interrogated in a reflection arrangement [18]. In this configuration, the changes in the reflected power indicate the SRI changes. Methods based on transmission or reflection measurements are sensitive to even small changes in the intensity of source radiation. Additional errors may also result from, for example, changes in the position of the optical fiber. Another parameter, correlated with SRI changes, which is determined on the basis of the TFBG spectrum, is the standard deviation estimator [19], expressed by the following equation:

(3) $$SD=\sqrt{\frac{1}{N-1}\sum_{i=1}^{N}{\left(T_{i}-\bar{T_{i}}\right)}^{2}}$$

It differs from the classical estimator, because $\bar{T_{i}}$ is not an average value; it is the spectrum smoothed using the Savitzky–Golay method. Therefore, the value is not the whole transmission spectrum average value, but rather the local average value without the cladding mode peaks. The problem of averaging a spectrum to remove peaks and the local average value spectrum will be presented in the following parts of the article.

Recently, the use of a parameter of the spectral contour length in the spectral range of the cladding modes for the determination of the SRI has been proposed [12]. It is the simplest parameter possible, because it does not require the calculation of spectral peaks or the smoothing and averaging of filters. The contour length can be calculated as follows:

(4) $$L=\sum_{i=1}^{N}\left|T_{i+1}-T_{i}\right|$$

For TFBG, pre-processing methods are rarely used. One example of a pre-processing method is the discrete wavelet transform (DWT), which is used to reduce noise before determining the wavelength shift of the individual modes. However, this method is used specifically for the tilted Moiré fiber Bragg grating (TMFBG) [20].

## 3. Experimental Measurements

The light from the super-luminescent diode S5FC1005S (Thorlabs Inc., Newton, NJ, USA) was directed to the TFBG inscribed in the hydrogen-loaded single-mode optical fiber. The signal was measured after passing through the entire system using the optical spectrum analyzer AQ6370D (Yokogawa, Tokyo, Japan). The measurements were carried out at a stabilized ambient temperature of 20 °C and at a constant SLD current of 200 mA. All the spectra analyzed here were measured with 0.02 nm resolution. The TFBG had a 6° tilt angle and was inscribed by the phase mask method using an excimer laser (Coherent Inc.). The grating was immersed in a water solution of glucose (minimum SRI = 1.333 pure water, maximum SRI = 1.4651 glucose 70%).

**Figure 2:**
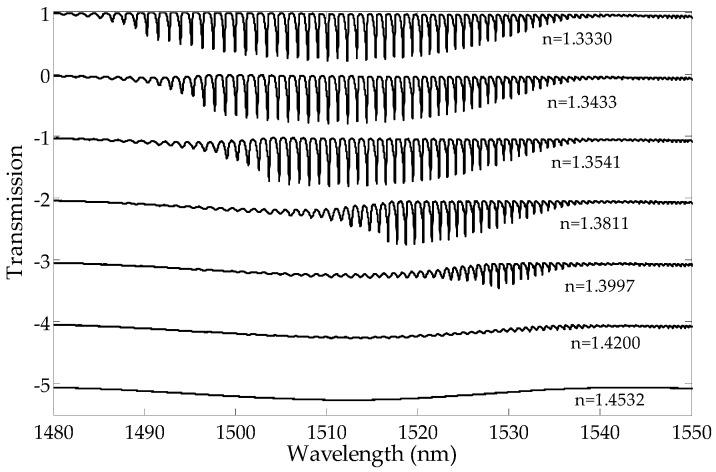
Measured TFBG transmission spectra for a few surrounding refractive indexes.

The transmission intensity of the higher order modes decreases with the increase in the SRI. In Figure 2, it can be observed that the amplitudes of the cladding modes for the shorter wavelengths are reduced first. The transmission intensity of the lower order modes does not change until the SRI reaches a certain value. For each resonance, there is an SRI value for which the mode is leaking from the optical fiber and its transmission peak intensity in the spectrum disappears. As a consequence, the transmission spectrum becomes smoothed out, and there are no visible peaks in the cladding modes. The cut-off wavelength is the wavelength after which a given mode is no longer propagated on the interface between the cladding and the surrounding media. The mode at the cut-off limit strongly shifts towards longer wavelengths while reducing its intensity. The leaking mode strongly reduces its intensity with a slight wavelength shift [21].

The peaks in the individual cladding modes in the spectrum are not evenly distributed. Near the Bragg peak, the distance between the resonances is 400 pm, but for smaller wavelengths the distance increases to 1000 pm [6]. The position of each resonance in the spectrum, which is associated with the propagation of a given mode, depends on its effective refractive index. Increasing the SRI causes a slight shift in each cladding resonance towards longer wavelengths. Increasing the SRI causes the mode to propagate deeper into the surrounding medium.

## 4. New Proposed Methods and Comparison with Existing Methods

The disadvantage of methods based on the envelope calculation is the need to determine the envelope, which involves finding the peaks for each mode. In this way, although the whole spectrum is processed, only a few points of the spectrum are taken into account in the algorithm. Therefore, methods based on variance and contour length that use all the spectrum points in the cladding mode range would be expected to be more reliable, because additional information from redundant wavelength measurements gives the advantage of noise reduction. In this article, we propose the use of a new parameter called mean deviation from the local mean, which is calculated using the following equation:

(5) $$MD=\frac{1}{N}\times \sum_{i=1}^{N}\left|T_{i}-{\bar{T}}_{l}\right|$$

**Figure 3:**
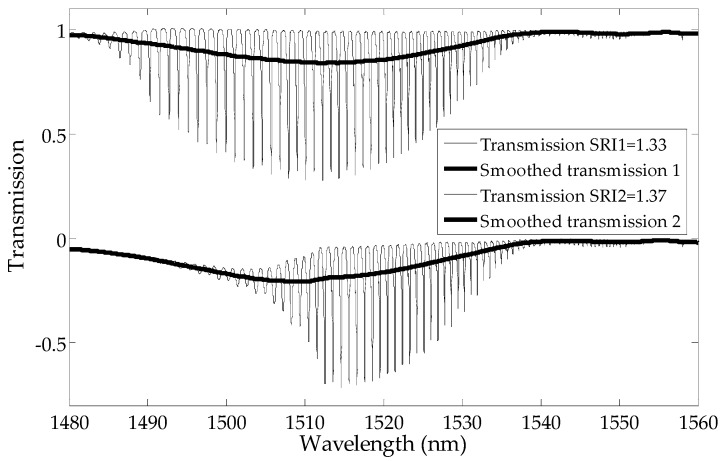
Two examples of transmission spectra and their smoothed versions (local averages).

We also propose a universal parameter, which is a group of parameters:

(6) $$UP={\left(\frac{1}{N}\times \sum_{i=1}^{N}{\left|T_{i}-{\bar{T}}_{i}\right|}^{p}\right)}^{q}$$

where ${\bar{T}}_{i}$ is the local average value of the transmission, which is calculated by the moving average filter (a rectangular filter kernel). The filter is then used to remove the components associated with the cladding mode peaks from the entire spectrum (Figure 3). Proper results can be obtained by using triangular moving averages with the filter length covering 3–4 maxima of cladding modes.

The moving average filter is the simplest of the finite impulse response filter. It is just the unweighted mean of previous samples for the causal filter. However, causal filters introduce a delay, which for the spectrum signal processing is manifested by its shift. Therefore, we ultimately use non-causal filters. These filters calculate the output signal based on past, current and future inputs. In principle, any filter that has smoothed cladding mode peaks is sufficient for this application. It is important that the filtered spectrum look similar to the spectrum measured for large SRIs, where all the modes have leaked from the structure outside the cladding. The subtraction of the local mean component from each point of the spectrum (Equation (5)) results in the removal of slowly changing components and leaves only the values related to the cladding modes resonances. Information about the SRI value is contained exactly in the changing shape of the individual peaks. The choice of *p* and *q* parameters of the UP algorithm (Equation (6)) allows algorithmic shaping of the sensor processing characteristics. The choice between algorithms and parameters can be based on the analysis of the linearity of the sensor processing characteristics. In our case, the nonlinearity is 8.6% for the mean deviation (MD), 7.8% for envelope, 7,1% for contour and 15.7% for SD in the 1.333–1.42 SRI range. The nonlinearity is 8.5% for MD, 9.2% for envelope, 7.7% for contour and 4.1% for the universal parameter (UP) (*p* = 0.75, *q* = 4/3) in the 1.333–1.4 SRI range.

**Figure 4:**
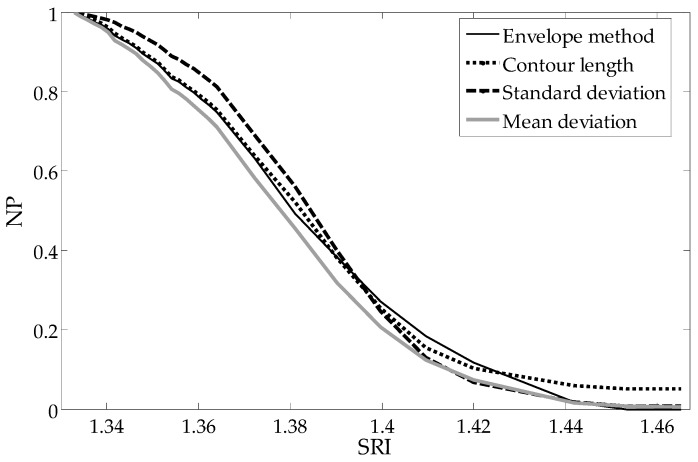
Comparison of calculated normalized parameters (NP) as a function of the surrounding refractive indexes.

To compare the shapes of the dependence of particular parameters from the SRI, each parameter is normalized to one representing its maximum value (pure water SRI = 1.333). The most important advantages of such global methods are the linear characteristics in a large part of the measurement range, which results in more even sensitivity of the sensor. The reduction of sensitivity, as well as non-linearity, only occurs for the most extreme SRI values being measured. As can be seen in Figure 4, the mean deviation from the mean algorithm results in a curve representing the processing characteristics that is shaped similarly to those of other popular methods.

The individual cladding modes have various effective refractive indexes (ERIs). If the ERI is lower than the SRI for a given mode, then the given mode leaks from the optical fiber. If the ERI is higher than the SRI for the specific mode, then the mode is guided in the optical fiber, because the signal associated with this mode is reflected according to the total internal reflection between the cladding and the surrounding medium. The mode for which ERI = SRI sets the cut-off border. Interestingly, modes that are close to the cut-off are not sensitive to polarization. Thus, they can also be used as parameters for indicating the SRI value [22].

Methods of determining the cut-off wavelength have very high sensitivity. However, due to the discrete distribution of the cladding modes at the wavelength, the processing characteristics are not continuous. Since the individual cladding modes are not evenly distributed over the wavelength, the sensitivity is not the same for the whole SRI range. Hence, an algorithm that uses both coarse and fine scales has been proposed and discussed [21]. The cut-off wavelength is indicated, therefore, by the location of a specific mode with decreasing intensity.

The cut-off wavelength can be determined by calculating the maximum value of the derivative of the lower and upper envelope, created by the cladding modes [8]. These methods make use of local changes near the spectrum place, where changes in the intensity of the modes occur. We propose a completely different approach, which consists of determining the curve of the cumulative value of the selected parameter (MD, UP) over the entire spectrum. The cumulative mean deviation (CMD) from the local mean over a wavelength of the TFBG spectrum can be defined as follows:

(7) $$CMD(k)=\frac{1}{k}\times \sum_{i=1}^{k}\left|T_{i}-{\bar{T}}_{i}\right|.$$

**Figure 5:**
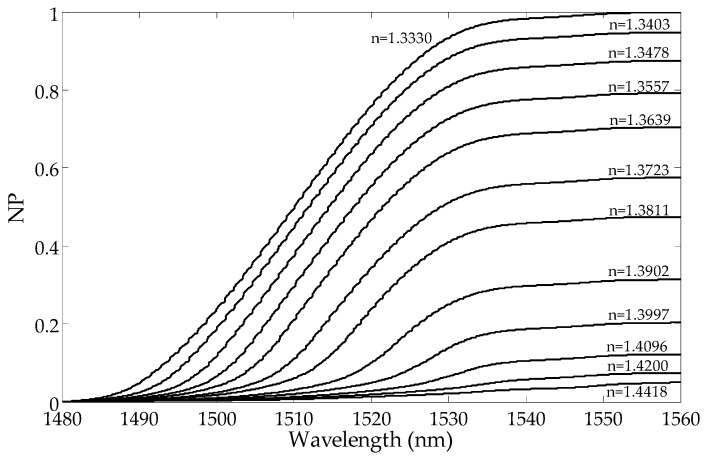
Changes in the normalized cumulative mean deviation from the local mean with the wavelengths for a few SRIs calculated from the side with shorter wavelengths.

**Figure 6:**
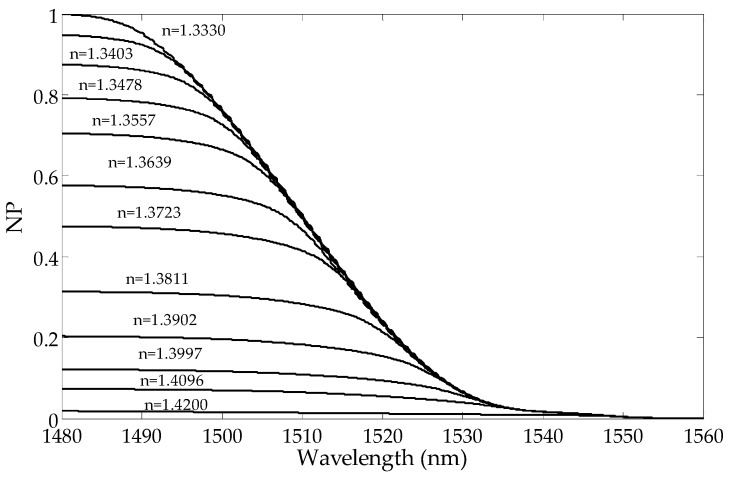
Changes in the normalized cumulative mean deviation from the local mean with the wavelengths for a few SRIs calculated from the side with longer wavelengths.

Unlike MD, which is the sum of the local differences in the spectrum from the local mean in the whole wavelength range, CMD is the sum of the differences on a given part of the cladding mode wavelength range. To calculate the cut-off wavelength, the CMD curve intersects with a horizontal reference curve, indicating the exact place where the mode starts to leak. Figure 5 and Figure 6 show curves of the cumulative change of the CMD parameter values along with the wavelengths. In typical application, the cumulative parameter calculated from the side with shorter wavelengths should be used. CMD calculated from the longer wavelength side can be more useful if SRI >1.39.

**Figure 7:**
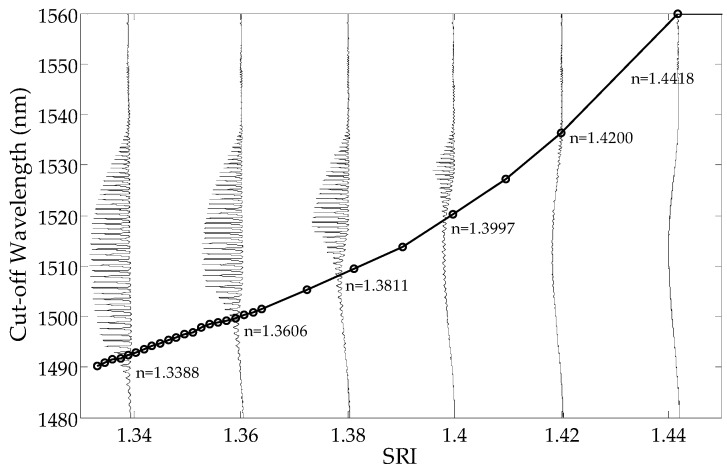
The cut-off wavelength calculated for different SRIs by the method of changing the cumulative mean deviation (CMD) parameter.

**Figure 8:**
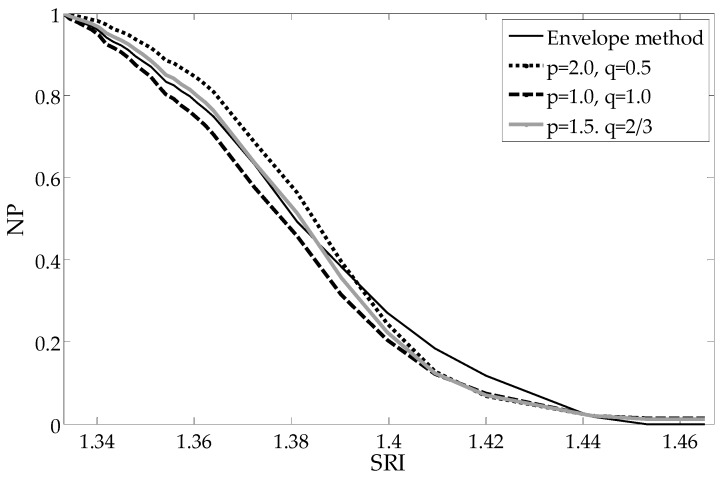
Comparison of the calculated NP versus the surrounding refractive index (SRI) for the universal parameter algorithms.

**Figure 9:**
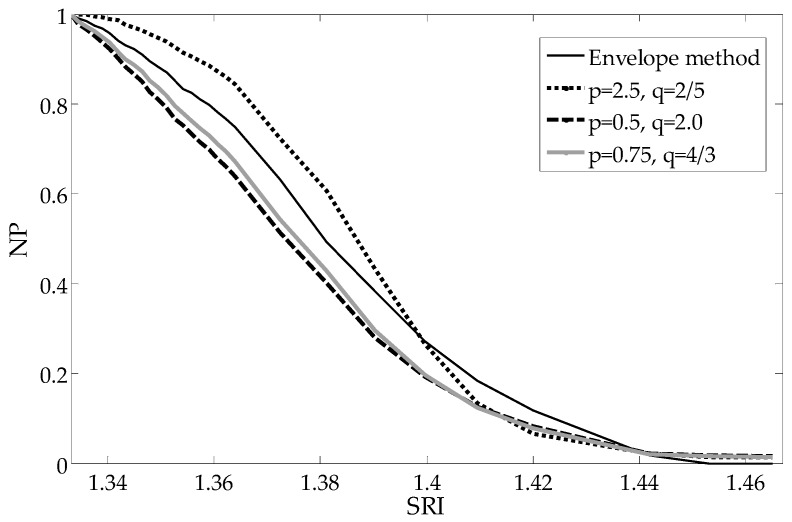
Comparison of the calculated NP versus the SRI for the universal parameter algorithms.

**Figure 10:**
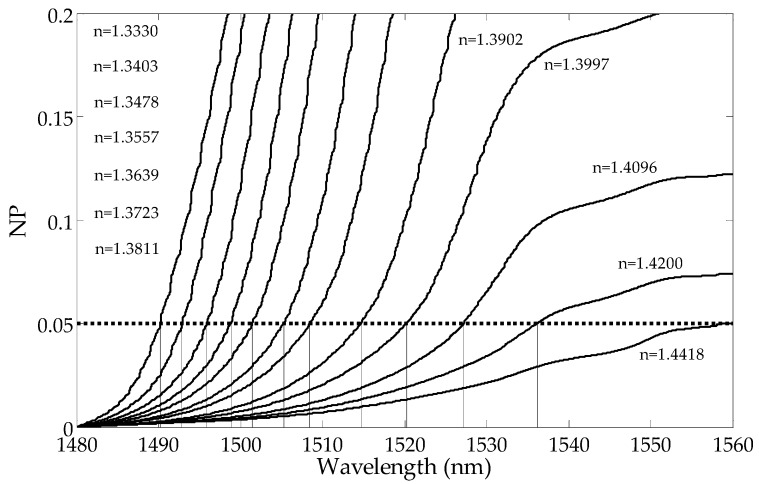
The method of cut-off wavelength calculation for different SRIs.

The curve representing dependence of the cut-off wavelength from the SRI is presented in Figure 7. As can be seen, the processing characteristics have good linearity. In comparison with direct global methods (Figure 4, Figure 8 and Figure 9), there is no significant non-linearity above 1.4 SRI. The calibration curve in Figure 10 was created by using the curves from Figure 7 and by finding a place at the wavelength of their intersection with a horizontal curve of 0.05, in the normalized parameter (NP) value (Figure 10). The results for the CMD and cumulative UP are similar, so we recommend using the MD parameter.

In order to compare both the existing and newly proposed methods, 500 repeated measurements were made for an SRI value of 1.3639. The obtained measurement resolution values are summarized in Table 1. The resolution was estimated using the calculated standard deviation and sensitivity of a series of measurements according to the method described in [11,23].

The most accurate method is based on the cut-off wavelength determination. The simplest method is the contour length algorithm. After the analysis of literature and the results obtained in this article, we suggest using the MD method in typical applications, the contour length for coarse measurements and the cut-off method for precise application. 

## 5. Conclusions

The application of TFBG as an SRI sensor requires the use of appropriate spectrum demodulation algorithms. The sensor calibration curve is the relationship between the SRI and spectrum changes represented by a specific designated parameter (extracted spectrum features) from the spectrum. The calibration curve must be determined for each grating separately. This paper presented both existing and new methods of determining SRI based on the TFBG spectrum. As a result of the spectrum analysis, new methods and modifications of existing ones were proposed. The first proposed method is a group of algorithms based on the deviation from the local mean (mean deviation being the simplest example) parameters as a direct indication of the SRI. The second proposed method utilizes the shape of the cumulative function of the parameters along the wavelength to estimate the cut-off wavelength. The proposed methods were evaluated and compared in terms of measurement resolution. The best resolution is provided by the methods relying on the indirect estimation of the SRI based on the cut-off wavelength.

## Figures and Tables

**Table 1:** Comparison of the TFBG spectrum demodulation algorithms.

Method.	Measurement Resolution
Envelope	1.7 × 10^−4^
Contour length	1.1 × 10^−4^
Mean deviation	4.9 × 10^−5^
Standard deviation	4.5 × 10^−5^
Cut-off method based on mean deviation	2.5 × 10^−5^
